# hBM-MSC-Laden 3D Bioprinted Gelatin–Alginate Hydrogels: Physicochemical Characterisation and Osteogenic Lineage Commitment

**DOI:** 10.3390/gels12050387

**Published:** 2026-05-01

**Authors:** Devy F. Garna, Zetian Zhang, Lucy Di-Silvio

**Affiliations:** 1 Centre for Craniofacial and Regenerative Biology, Faculty of Dentistry, Oral and Craniofacial Sciences, King’s College London, London SE9 1RT, UK; 2 Centre for Oral, Clinical and Translational Sciences, Faculty of Dentistry, Oral and Craniofacial Sciences, King’s College London, London SE9 1RT, UK; zetian.zhang@kcl.ac.uk

**Keywords:** gelatin–alginate hydrogels, 3D bioprinting, mesenchymal stem cells, osteogenic differentiation, bone tissue engineering

## Abstract

Gelatin–alginate composite hydrogels are some of the most prevalent bioinks used for extrusion-based three-dimensional (3D) bioprinting because of their combined bioactivity and ability to ionically crosslink. Ionically crosslinked gelatin–alginate constructs containing human bone marrow–derived mesenchymal stem cells (hBM-MSCs) were characterised over time under standardised in vitro conditions to assess physicochemical properties and resultant cell behaviour. Water uptake and degradation were quantified over time in phosphate-buffered saline (PBS) and collagenase type II media for up to 21 days. Cell viability and metabolic activity were quantified, and osteogenic gene expression (RUNX2, COL1A1, OCN) was assessed. Raman spectroscopy and compressive mechanical characterisation were performed. Collagen and glycosaminoglycan-related peaks were observed from extracellular matrix (ECM)-associated components, with an increased presence of protein-associated signatures later in culture. Hydrogels displayed nonlinear elastic behaviour with increased stress after longer incubation times, suggesting no degradation of mechanical integrity over the duration of the study. Hydrogels experienced rapid hydration followed by decreased swelling over time, with a maximum swelling ratio at 24 h. Degradation rates significantly increased over longer incubation times (*p* < 0.001) and in collagenase media compared to PBS (*p* < 0.001). Observed differences were likely due to both ion-exchange-mediated network disassembly and the dissolution of gelatin components. Cell metabolic activity decreased under osteogenic culture conditions, while changes in osteogenic marker expression were sequential, suggesting a transition from proliferation to early osteogenic commitment in this 3D system. This work provides both physicochemical and biological characterisation of a commonly utilised gelatin–alginate bioink system, to provide future optimisations within the field of extrusion-based bone tissue engineering, a reproducible baseline for future optimisation of bioink systems in extrusion-based bone tissue engineering.

## 1. Introduction

Bone defects and injuries are a major clinical problem since the regenerative capacity of bone tissue is limited. In such scenarios, the injury’s complexity often exceeds the regenerative capacity, and surgical intervention is required [1,2]. The conventional approaches to bone repair include autografts, allografts, and synthetic bone graft substitutes; however, these technologies have faced major limitations, including donor site morbidity, tissue availability, immune rejection, disease transmission, and a lack of biological guidance for tissue regeneration [2,3,4]. Thus, tissue engineering approaches that integrate biomaterials, stem cells, and 3D printing have been explored to develop patient-specific bone substitutes that may potentially improve tissue regeneration [4,5,6].

Three-dimensional (3D) bioprinting is one such technology that has gained significant attention for tissue engineering applications over the last ten years, owing to its potential to develop complex tissue constructs with spatially controlled cell and biomaterial organisation [7,8]. Extrusion-based 3D bioprinting is a technology that allows for the additive manufacturing of 3D hydrogel scaffolds, primarily composed of cell-laden hydrogels. The cell-laden hydrogels are often formulated from biomaterial formulations, termed ‘bioinks’, that can act as scaffolds for cell encapsulation [9,10]. The printability, mechanical properties, and cytocompatibility, as well as the potential to promote cell survival and functionality, are often attributed to the physicochemical properties of the designed bioink formulation.

Gelatin–alginate hydrogels are commonly used in extrusion-based 3D bioprinting and have been widely investigated for tissue engineering applications [11]. The first reported 3D printing of living tissue constructs using a cell-assembly approach, where cells are directly stacked with a hydrogel biomaterial, appeared in [12]. Subsequent works have exploited this idea to print viable constructs of hepatocytes and other cell–biomaterial combinations [13,14,15]. Other groups have explored the use of gelatin-based composite hydrogels to create physiologically relevant tissue constructs and to influence the differentiation of encapsulated cells within a 3D environment [16,17]. Immediately after the seminal works on the use of gelatin-based hydrogels as a viable biomaterial for cell-based bioprinting applications, Wang et al. published a review article on the properties and uses of gelatin-based hydrogels in organ bioprinting [18].

Even though more advanced bioink systems exist, such as gelatin methacryloyl (GelMA), decellularised extracellular matrix (dECM) bioinks, and nanoparticle-reinforced hydrogels, ionically crosslinked gelatin–alginate systems are still widely used because they are cheap, easy to make, and have established cell compatibility of the materials [5,6,18]. Gelatin provides cells with Arg-Gly-Asp (RGD) sequences required to influence cell adhesion and spreading on a surface via integrin binding [19,20]. On the other hand, sodium alginate readily crosslinks into a hydrogel biomaterial via ionic crosslinking with divalent cations (Ca^2+^), which provides structural stability and shear-thinning properties of the biomaterials [21,22]. They can be readily formulated with shear-thinning properties suitable for extrusion-based deposition and rapidly gel after printing to create hydrated 3D matrices for cell viability assessments [7,23].

Hydrogel biomaterial properties also play a significant role in the biological performance of the resulting bioprinted scaffolds. Hydrogel hydration, a measure of the water content the hydrogel can retain, has been used to assess its water-retention capacity and, in turn, the nutrient, oxygen, and permeability properties of the hydrogel [24]. Another important physicochemical property of a biomaterial is its degradation rate, which is measured by the rate at which the scaffold is degraded. New extracellular matrices are produced to ensure that the structural integrity is maintained [19,25,26]. The degradation properties of ionic crosslinked gelatin–alginate hydrogels involve a change that results from the exchange of ions, where the divalent crosslinking ions are replaced, and the gelatin chains dissolve into the surrounding medium [20,21]. The degradation properties may also affect the mechanical properties of the hydrogel, and this may affect the behaviour of the cells that are encapsulated [27,28].

Human bone marrow–derived mesenchymal stem cells (hBM-MSCs) are commonly used cells for bone tissue engineering because these cells are capable of differentiating into an osteogenic lineage when subjected to mechanical and biochemical stimuli [29]. The process of differentiating into an osteogenic lineage begins with the upregulation of RUNX2, a master regulator that is responsible for the differentiation of the cells into an osteoblast lineage [30]. The next step is the upregulation of COL1A1 to denote the production of collagen, followed by the expression of osteocalcin (OCN) [31], which is correlated with the extracellular matrices that are produced. Temporal evaluation of this RUNX2 → COL1A1 → OCN transcriptional pathway in 3D hydrogel culture will offer insights into the process of osteogenic differentiation at the molecular level [32].

Although gelatin–alginate hydrogels are widely used for extrusion-based 3D bioprinting techniques, very few studies have established the relationship between hydrogel properties and cellular response under standardised testing conditions. Moreover, hydrogel properties such as water uptake and degradation have never been directly correlated with cell viability and osteogenic gene expression in hBM-MSC-laden hydrogels. Here, we will evaluate the properties of hydrogels used in our study for 3D bioprinting applications using bioink consisting of 3% *w*/*v* gelatin and 5% *w*/*v* sodium alginate. We have already optimised and validated this bioink for our laboratory conditions and have been successful in using this bioink to fabricate a 3D bioprinted biphasic scaffold for tissue engineering at the bone–cartilage interface [33]. The water uptake kinetics of our cell-laden bioink were evaluated over time. The in vitro degradation of the hydrogel was also performed using both PBS and collagenase type II. The cell viability and metabolic activity were assessed under both control and osteogenic conditions. Osteogenic gene expressions of RUNX2, COL1A1, and OCN were also performed over a 21-day culture period in hBM-MSC-laden hydrogel. Raman spectroscopy was used to evaluate the biochemical composition of hydrogel constructs. Compressive mechanical testing was performed to evaluate the macrostructure of hydrogels. This information will provide insights into the properties of hydrogels used in our study, and the information will be useful to others to evaluate the properties of hydrogels used for extrusion-based bioprinting techniques.

## 2. Results and Discussion

### 2.1. Degradation and Swelling Rate

**Figure 1:**
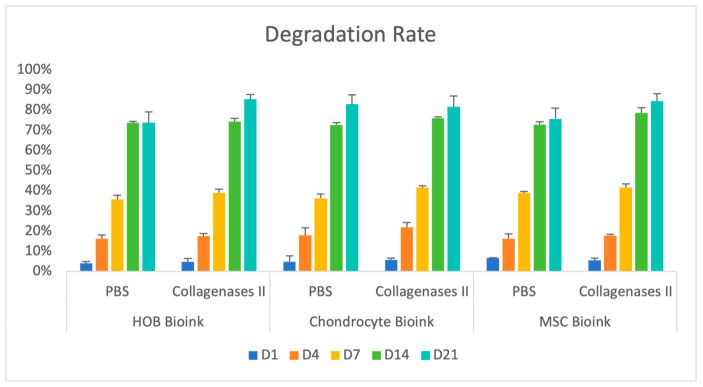
Degradation rate (%) of 3D bioprinted gelatin–alginate hydrogels incubated in PBS or collagenase type II over 21 days (D1, D4, D7, D14, and D21). Data are presented as mean ± SD (*n* = 3). Statistical analysis was performed using three-way ANOVA to assess the effects of degradation medium, bioink formulation, and time, followed by Bonferroni post hoc testing where appropriate.

Three-way ANOVA demonstrated a significant main effect of degradation medium (F(1,60) = 28.099, *p* < 0.001, partial η^2^ = 0.319), indicating greater mass loss under collagenase conditions compared with PBS. Culture duration significantly influenced degradation (F(4,60) = 3212.613, *p* < 0.001, partial η^2^ = 0.995), confirming progressive time-dependent mass loss across all groups. In contrast, gel formulation alone did not significantly affect degradation (F(2,60) = 1.805, *p* = 0.173) (Figure 1). A significant three-way interaction among degradation medium, gel type, and time was observed (F(8,60) = 2.142, *p* = 0.045, partial η^2^ = 0.222), indicating that time-dependent degradation patterns varied modestly among bioink formulations under different incubation conditions. However, Bonferroni post hoc comparisons did not reveal statistically significant differences between gel types at corresponding time points (*p* > 0.05).

**Figure 2:**
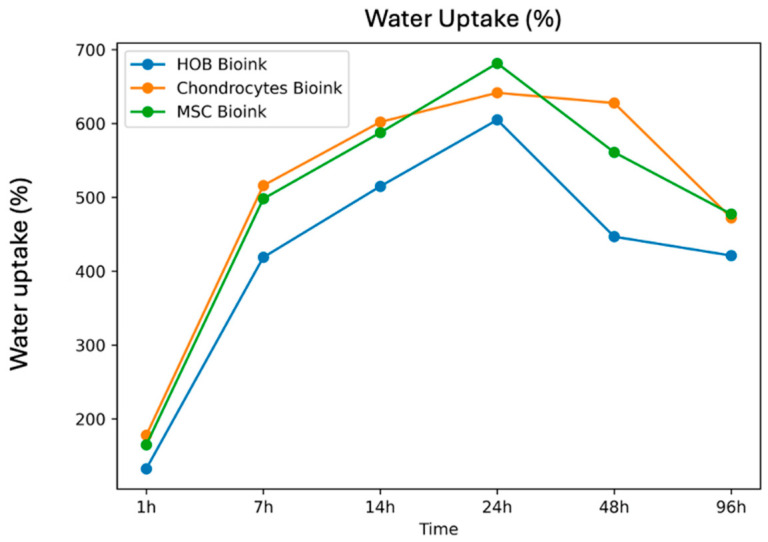
Swelling behaviour of cell-laden bioinks over time. Swelling degree (%) of HOB-, chondrocyte-, and MSC-laden bioinks measured at 1, 7, 14, 24, 48, and 96 h. All groups exhibited rapid initial swelling within the first 7 h, reaching peak swelling at 24 h, followed by a gradual decrease. Data are presented as mean values. Statistical analysis was not performed due to the absence of replicate-level raw data.

The swelling behaviour of the three bioink formulations showed rapid water uptake during the first 24 h, followed by stabilisation or a gradual decrease at later time points (Figure 2). The HOB bioink increased from 132.49% at 1 h to a maximum of 604.89% at 24 h, then decreased to 421.10% at 96 h. The chondrocyte bioink reached a maximum water uptake of 641.60% at 24 h and remained comparatively elevated at 48 h (627.68%), before decreasing to 472.17% at 96 h. The MSC bioink showed the highest observed peak water uptake (681.50% at 24 h), followed by a reduction to 477.36% at 96 h. These observations indicate pronounced early hydration across all formulations, with peak water uptake observed at ~24 h. These measurements are presented as reported summary values.

Replicate-level raw data (e.g., individual sample values and dispersion metrics) were not available for this dataset; therefore, no inferential statistical analyses were performed for swelling. This limitation reflects data availability rather than experimental design. These data are presented to provide descriptive insight into hydration behaviour rather than comparative statistical analysis.

The degradation behaviour and water uptake properties of the bioinks were evaluated by measuring mass loss over time. All hydrogel formulations degraded progressively under both PBS and collagenase treatment conditions over the course of the 21-day culture period. Statistical analysis revealed main effects of incubation medium and timepoint on degradation but not gel type (Figure 1). These data suggest that both bioinks followed similar degradation trajectories, primarily controlled by physicochemical processes associated with the gelatin–alginate hydrogel network rather than differences between cell-laden hydrogels.

Alginate–gelatin hydrogels crosslinked ionically are subject to several degradative processes that occur simultaneously over time. In PBS, Ca^2+^ crosslinks within the alginate network can be replaced over time by monovalent cations present in the PBS solution (Na^+^, K^+^), resulting in decreased crosslink density and gradual loss of hydrogel mass independent of collagenase treatment [21,22]. Degradation can also occur via dissolution of gelatin molecules within the hydrogel network. At 37 °C, gelatin approaches its gel-to-sol phase transition temperature, allowing uncrosslinked or weakly interacting gelatin polymers to slowly diffuse into the surrounding medium [20,22]. Because gelatin molecules are physically rather than covalently trapped within the hydrogel network, collagenase treatment will not result in complete hydrogel dissolution. Instead, polymer entanglements and alginate–gelatin ionic crosslinks can sterically limit collagenase accessibility to gelatin chains [22,34]. Thus, ion exchange with PBS and passive gelatin dissolution provide possible explanations for why degradation behaviour was similar between PBS and collagenase-treated samples.

Despite finding no statistical effect of gel formulation, there was a significant three-way interaction between incubation medium, gel type, and time. A similar trend has been observed in gelatin–alginate hydrogels, where cell-laden constructs displayed slightly different degradation rates over time [34,35]. Although mass loss data were normalised to day 0 measurements, these data do not account for increases in hydrogel mass associated with water uptake. As such, changes in hydrogel weight over time likely reflect the combined effects of water incorporation and degradation.

Immediately following hydrogel synthesis and gelation, all three bioink formulations experienced rapid water uptake, achieving maximum swelling by 24 h before plateauing or declining until 96 h post-printing (Figure 2). Rapid swelling of the hydrogel network is attributed to the hydrophilic nature of alginate carboxylate groups and gelatin residues [21,36]. The decrease in swelling that follows the initial water uptake phase can be attributed to physicochemical changes within the hydrogel. Gelatin dissolution decreases the overall count of hydrophilic polymer chains within the construct [21,37]. Loss of gelatin into the surrounding medium further reduces hydrophilicity and hydrogel water-retention capacity [20,36]. Gelatin–alginate constructs have also been shown to exhibit this swelling pattern [36,37]. Since replicate-level raw data were unavailable for swelling measurements, data are presented descriptively. Nonetheless, the swelling behaviour we observe is similar to previously-reported hydration patterns and can be expected of hydrophilic polymer networks [38,39].

### 2.2. Cell Viability

**Figure 3:**
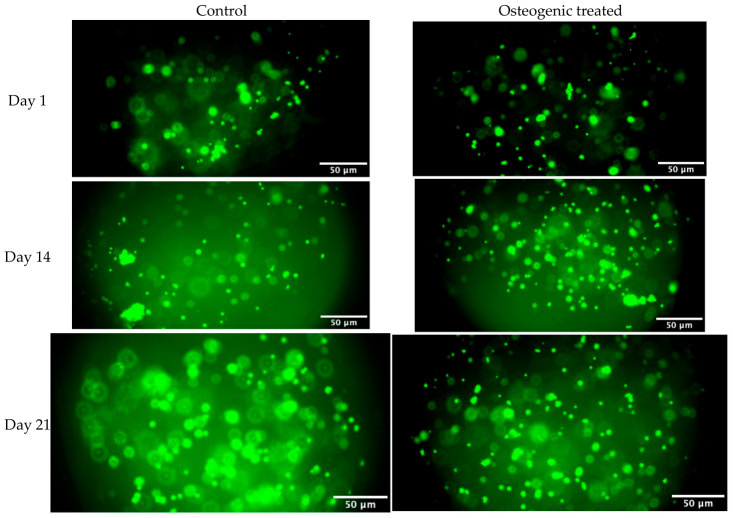
Representative Live/Dead fluorescence images of constructs cultured under control or osteogenic medium at Days 1, 14, and 21. Green fluorescence indicates live cells. Images are representative of independent samples. Scale bars: 50 μm.

Cell viability was evaluated using Live/Dead staining at Days 1, 14, and 21 (Figure 3). Predominant green fluorescence was observed in both control and osteogenic groups at all evaluated time points, indicating high cell viability. An increase in cell density was evident over time, with no observable reduction in viability under osteogenic conditions. These findings indicate that osteogenic induction did not adversely affect cell survival throughout the 21-day culture period.

**Figure 4:**
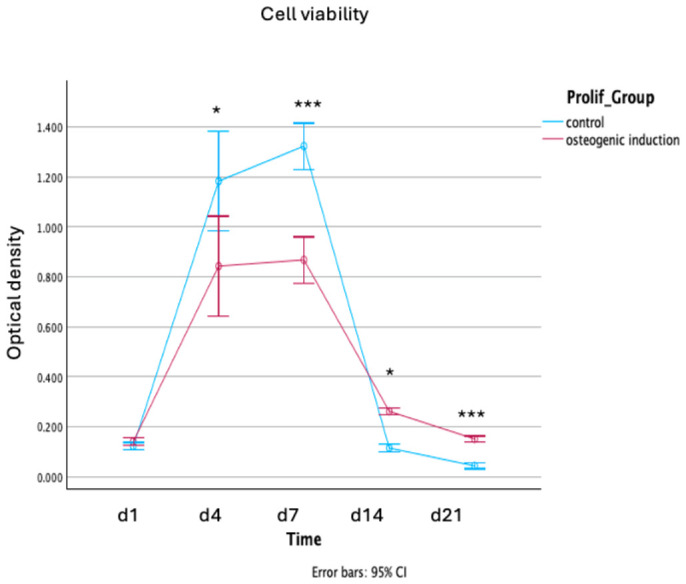
Longitudinal metabolic activity of cells within hydrogel constructs over 21 days. Comparison between control (blue) and osteogenic induction (maroon) groups. Data are presented as estimated marginal means ± 95% confidence intervals. Statistical analysis was performed using a two-way mixed ANOVA with Greenhouse–Geisser correction. Post hoc comparisons were conducted using the Games–Howell test. Significance levels: * *p* < 0.05, *** *p* < 0.001.

The proliferation of cells within the hydrogel scaffolds was monitored over 21 days (Figure 4). A two-way mixed ANOVA revealed a highly significant interaction between Time and Treatment Group (Greenhouse–Geisser corrected: F(1.26,12.62) = 17.67, *p* < 0.001, ηp^2^ = 0.639), indicating that the two groups followed significantly different proliferation trajectories.

All samples showed an initial increase in cell viability. The control group exhibited a rapid expansion phase, peaking at Day 7 with significantly higher proliferation compared to the osteogenic induction group (*p* < 0.001). This phase was characterised by a transient increase in variance at Day 4, reflecting greater heterogeneity in cell viability across replicates.

The control group showed a sharp decline in viability at Day 14 and Day 21. Conversely, the osteogenic induction group maintained a significantly more stable proliferation profile. By Day 21, the osteogenic group demonstrated significantly higher sustained viability than the control (*p* < 0.001), suggesting that while the induction factors initially slow proliferation to favour differentiation, they ultimately provide a more sustainable long-term environment for cell survival.

Microscopy images acquired from Live/Dead assays showed prominent green staining in both control and osteogenic groups at all time points (Figure 3). This staining pattern indicates maintenance of cell viability across the duration of the culture period. There was a gradual increase in cell density over time without any apparent decrease in viability during osteogenic induction. These results suggest that osteogenic supplements added to the media did not induce cytotoxic effects at the concentrations used, consistent with a previous report [40]. In agreement with earlier studies, gelatin–alginate hydrogels allow long-term survival of MSCs after osteogenic induction [34,41].

A limitation of the Live/Dead assay in this study is that fluorescence images were acquired as single focal-plane micrographs rather than stitched images representing the full thickness of each construct. Images presented in Figure 3, therefore, only serve as a representative example of viable cells near the focal plane. Implementation of confocal tile-scans or cross-sectional analysis at future timepoints will allow for more comprehensive visualisation of cells throughout the interior of the printed constructs.

The metabolic activity assay shown in Figure 4 reports significantly lower metabolic activity in osteogenic samples relative to control groups at later culture timepoints. It is common for MSCs to exhibit reduced metabolic activity after osteogenic induction in vitro [30,42,43]. During osteogenic differentiation, MSCs transition from cellular proliferation towards extracellular matrix deposition and terminal differentiation. This reduces activity on assays such as alamarBlue, which measure cellular metabolites associated with glycolysis [42]. Thus, reduced signal over time in osteogenically induced samples likely does not represent cell death but instead downregulation of the metabolic pathways detected by this assay during osteogenic differentiation. The Live/Dead assay results serve as a useful confirmation of this interpretation by showing that cell viability does not decrease over time in osteogenically induced samples.

The high standard deviation seen in the Day 4 control group is another limitation of this dataset. This phenomenon could be biological, corresponding to increased heterogeneity in cell proliferation during early expansion before cells become more confluent. Regardless of cause, this transient increase in standard deviation does not affect the overall interpretation of the dataset because the group-time interaction term remained significant after Greenhouse–Geisser correction. Similar increases in cell population heterogeneity during early culture have been observed in 3D hydrogel cultures [44,45]. As cells grown in 2D monocultures typically proliferate at a consistent rate, diffusion limitations and local microenvironmental heterogeneity create mixed proliferative/quiescent/hypoxic cell populations in hydrogel scaffolds.

### 2.3. Morphological Evaluation by Scanning Electron Microscopy

**Figure 5:**
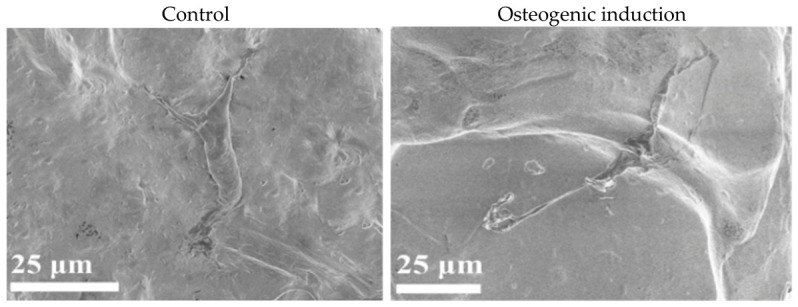
Scanning electron microscopy (SEM) micrographs of hBM-MSC-laden gelatin–alginate constructs at Day 7 cultured under control (growth medium) and osteogenic induction conditions. In the control group, cells exhibited a relatively flattened morphology with limited matrix deposition. Under osteogenic induction, cells displayed enhanced spreading and increased extracellular matrix-like coverage. Scale bars: 25 μm.

Scanning electron microscopy (SEM) was used to qualitatively assess hBM-MSC morphology within 3D bioprinted gelatin–alginate constructs after 7 days of culture under control (growth medium) or osteogenic induction conditions (Figure 5). In the control group, hBM-MSCs exhibited a relatively flattened and partially elongated morphology with limited evidence of extracellular matrix (ECM) coverage on the hydrogel surface. Cells appeared attached to the scaffold matrix but displayed minimal surface-associated deposition.

By contrast, osteogenically stimulated constructs showed more extensive cellular spreading and greater coverage by ECM-like material. Cells appeared more interconnected, with pronounced extensions along the hydrogel surface, consistent with active matrix remodelling within the scaffold network. These qualitative morphological differences suggest that osteogenic induction modulates early cell–matrix interactions in the 3D bioink microenvironment, although SEM alone cannot definitively confirm osteogenic differentiation.

The morphological differences observed here are consistent with increased expression of ECM proteins during early osteogenic differentiation [10]. However, without additional assays, these data do not prove that osteogenic differentiation occurred. As mentioned in the previous section, MSCs become more quiescent as they terminally differentiate during osteogenesis. Due to this, increased cell spreading and production of ECM molecules can occur through simple cell–matrix interactions over an extended culture period. Further characterisation using osteogenic staining (e.g., Alizarin Red S or Von Kossa staining), analysis of alkaline phosphatase activity, or detection of osteogenic marker proteins (osteocalcin, osteopontin, RUNX2) at the protein level would be necessary to confirm osteogenic differentiation [46]. These analyses were not performed in the present study and represent important directions for future investigation. The SEM results are, therefore, interpreted here as providing morphological context, indicating that hBM-MSCs remained adherent, spread, and metabolically active within the gelatin–alginate matrix.

### 2.4. Osteogenic-Related Markers Expression

**Figure 6:**
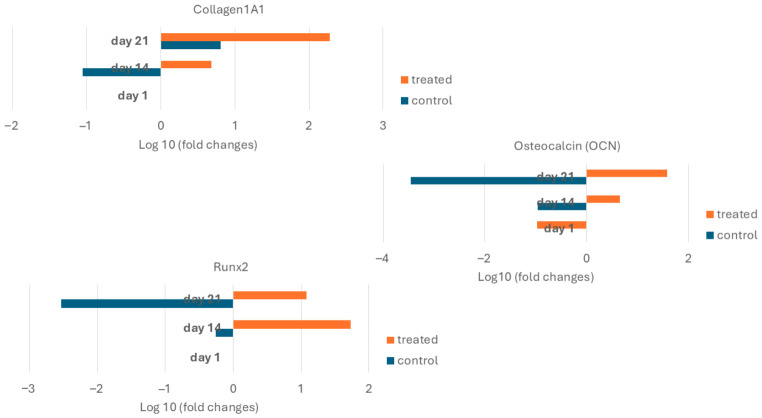
Osteogenic gene expression of hBM-MSC-laden constructs over time. Relative gene expression (log10 fold change) of COL1A1, RUNX2, and osteocalcin (OCN) at three time points: Day 1, Day 14, and Day 21 under control and osteogenic (treated) conditions. Expression levels are presented relative to baseline. Differences in gene expression across treatment conditions and time points were analysed using two-way ANOVA.

Two-way ANOVA demonstrated that osteogenic induction significantly influenced gene expression across all markers (Figure 6). COL1A1 expression was significantly increased under osteogenic conditions (F(1,48) = 9.63, *p* = 0.003), with a significant effect of culture duration (*p* < 0.001) but no interaction between treatment and time. For RUNX2, both treatment (F(1,48) = 37.44, *p* < 0.001) and time (*p* = 0.001) had significant effects, with a significant interaction (*p* < 0.001), indicating a time-dependent response to osteogenic stimulation. Similarly, OCN expression was significantly influenced by osteogenic treatment (F(1,48) = 48.67, *p* < 0.001), with a significant interaction between treatment and time (*p* < 0.001). In contrast, time alone was not significant (*p* = 0.069), suggesting that changes in OCN expression were primarily driven by osteogenic induction.

Based on established models of osteogenic differentiation, RUNX2 activation initiates osteoblast commitment, COL1A1 upregulation indicates active collagen deposition within the ECM, and OCN serves as a late-stage marker for matrix maturation [30,31]. We have observed similar trends when culturing hBM-MSCs-laden alginate-based bioprinted constructs under osteogenic conditions [34,47].

Nevertheless, several limitations of this gene expression dataset should be considered. First, the three markers analysed represent only a subset of the osteogenic transcriptional programme. Additional genes, including alkaline phosphatase (ALPL), osterix (SP7), bone sialoprotein (IBSP), and osteopontin (SPP1), would provide a more comprehensive evaluation of osteogenic differentiation [48]. Moreover, gene expression data alone cannot confirm functional osteoblast differentiation without corresponding protein-level validation or evidence of matrix mineralisation.

Secondly, while the hydrogel microenvironment likely plays a role in directing cell behaviour, the experimental design does not allow for decoupling of osteogenic medium from scaffold effects. All ingredients within the osteogenic medium (β-glycerophosphate, L-ascorbic acid, and dexamethasone) promote osteogenic differentiation of MSCs in culture. Without a 2D osteogenic control, it is impossible to know how much of the observed cell behaviour was due to the supplemented factors versus the effects of the gelatin–alginate hydrogel. Including 2D controls or parallel culture conditions without osteogenic supplements would help address this limitation in future studies.

Third, we selected an appropriate composition of gelatin–alginate hydrogel based on previous studies [33], which reported high cell viability and desirable physicochemical properties within a gelatin–alginate-based bioprinted biphasic scaffold designed for osteochondral interface tissue engineering applications. Here, we demonstrate the application of the same gelatin–alginate composition to culture hBM-MSCs under osteogenic conditions. However, an optimisation study characterising varying gelatin–alginate ratios was outside the scope of this study and represents an important area of future work.

Taken together, the expression trends shown in Figure 6 indicate molecular changes consistent with osteogenic lineage commitment of hBM-MSCs within gelatin–alginate hydrogels under osteogenic induction conditions. However, future work will be required to demonstrate complete osteoblast differentiation and ECM mineralisation

### 2.5. Ramanspectroscopic Analysis

Raman spectroscopy was used to assess biochemical changes within the 3D bioprinted hydrogel constructs over culture time. In the cell-laden bioink group, bands were observed at approximately 866 cm^−1^ and 1381 cm^−1^, which were assigned to collagen- and glycosaminoglycan (GAG)-associated signals, respectively. Additional features were observed in the 950–960 cm^−1^, 1080–1090 cm^−1^, and 1620–1640 cm^−1^ regions, corresponding to DNA-associated vibration, lipid-associated signal, and protein-associated signal, respectively.

**Figure 7:**
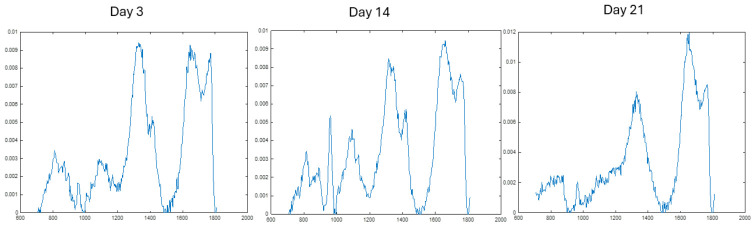
Raman spectra of cell-laden 3D bioprinted gelatin–alginate hydrogels at Days 3, 14, and 21. Spectra are presented within the selected wavenumber range (600–1800 cm^−1^) to highlight the most relevant biochemical features. Characteristic bands corresponding to collagen (~866 cm^−1^), glycosaminoglycans (~1381 cm^−1^), and protein (~1620–1640 cm^−1^) are indicated. Spectra were baseline-corrected, smoothed, and normalised. Representative spectra are shown.

Across the bioink samples, the protein-associated band at 1620–1640 cm^−1^ increased from Day 14 to Day 21, while collagen- and GAG-associated peaks remained detectable throughout the culture period. These findings suggest progressive biochemical maturation of the cell-laden constructs and are consistent with ongoing extracellular matrix deposition within the hydrogel (Figure 7).

Characteristic bands corresponding to collagen (~866 cm^−1^), glycosaminoglycans (~1381 cm^−1^), and proteins (amide I region, ~1620–1640 cm^−1^) were observed, indicating the presence of extracellular matrix (ECM)-associated components within the constructs [49,50,51]. This observation suggests that ECM-associated molecules are produced within the hydrogel constructs over time. The slight increase in intensity within the protein amide-I region at later time points may suggest increasing matrix production as the hydrogel matures. Interestingly, there is also a weak band within the range of 950–960 cm^−1^ that could correspond to phosphate vibrations of apatite [52,53,54]. This may suggest evidence of matrix mineralisation within hydrogels over time; however, this Raman spectrum was only reported qualitatively without quantitative analysis or complementary assays.

### 2.6. Mechanical Characterisation of Bioprinted Hydrogels

**Figure 8:**
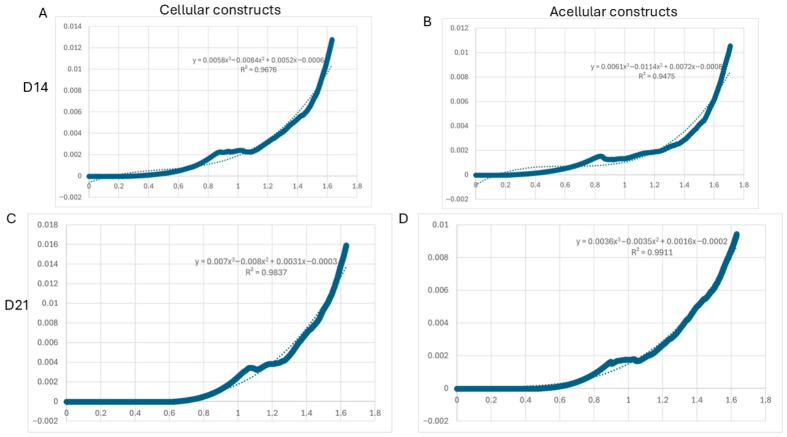
Compressive stress–strain behaviour of 3D bioprinted gelatin–alginate hydrogels over time. Representative stress–strain curves of cell-laden (**A**,**C**) and acellular (**B**,**D**) constructs at Day 14 (**A**,**B**) and Day 21 (**C**,**D**) under unconfined compression. All samples exhibited nonlinear elastic behaviour typical of hydrogel systems, with increased stress observed at higher strains. Solid lines represent the experimental data, while dotted lines indicate the corresponding polynomial trend lines used to illustrate the overall mechanical response. Curves are presented as representative measurements.

The compressive mechanical behaviour of the bioprinted hydrogels was assessed using unconfined compression testing (Figure 8). All samples showed nonlinear stress–strain responses typical of hydrated polymer networks, with an initial low-stiffness region followed by a gradual increase in stress at higher strain levels.

At Day 14, both cell-laden and acellular constructs exhibited similar mechanical profiles, with stress gradually increasing beyond approximately 0.8 strain. However, at Day 21, an overall rise in stress at comparable strain levels was observed, indicating improved structural integrity of the hydrogels over time.

Comparisons between cell-laden and acellular constructs revealed subtle differences in curve shapes, especially at higher strains, suggesting that the presence of encapsulated cells may influence the mechanical behaviour of the hydrogel matrix. Overall, these results indicate that the bioprinted constructs maintain structural stability and exhibit a time-dependent evolution in mechanical properties.

The compressive stress–strain curves for both hydrogel formulations are nonlinear elastic and display an initial linear region followed by strain stiffening [55,56,57]. At later timepoints, there is an increase in stress at a given strain value, suggesting increasing stiffness over time.

At lower strains, cell-laden hydrogels display decreased stress values compared to the acellular hydrogel controls. As strain increases, cell-laden hydrogels behave more similarly to their acellular counterparts. This difference in mechanical response between cell-laden and acellular hydrogels at higher strains could be due to cell encapsulation effects on matrix deposition. Previous studies have shown that hydrogel mechanics can vary depending on encapsulated cells [58,59,60].

Overall, these findings suggest that the bioprinted hydrogels maintain mechanical stability while undergoing time-dependent changes, although further quantitative analysis would be required to fully characterise stiffness variations between groups.

### 2.7. Scaffold Microenvironment and Mechanosensing

Beyond providing structural support, the gelatin–alginate matrix may influence the behaviour of encapsulated hBM-MSCs through mechanobiological mechanisms. Progressive degradation of the hydrogel network can alter matrix stiffness and porosity over time, potentially modulating cellular mechanosensing pathways. Changes in matrix mechanics have been shown to regulate MSC osteogenic commitment through integrin–focal adhesion kinase (FAK) signalling and downstream YAP/TAZ mechanotransduction pathways [61,62,63]. In addition, gelatin-derived RGD motifs facilitate integrin binding and cytoskeletal tension, which can promote RUNX2 activation through mechanosensitive signalling processes [33,62].

Although mechanotransductive pathways were not directly examined in this study, the sustained upregulation of RUNX2 during the culture period may be consistent with continued mechanosensory input from the evolving hydrogel microenvironment. Further work using pathway inhibitors or controlled matrix stiffness systems would be required to directly evaluate the role of mechanotransduction in regulating osteogenic differentiation within these constructs.

### 2.8. Limitations and Future Perspectives

This study provides a combined physicochemical and biological dataset for a commonly used gelatin–alginate bioink system; however, several limitations should be acknowledged. While compressive stress–strain testing was performed to evaluate the bulk mechanical properties of printed constructs, rheological characterisation of the bioink (including viscosity, shear-thinning behaviour, and viscoelastic parameters) was not conducted. As the present work focuses on post-printing physicochemical evolution and biological response, rheological assessment and printability optimisation were beyond its scope, particularly given that this formulation has been previously validated for extrusion-based bioprinting.

In addition, only a single bioink composition (3% gelatin/5% alginate) was investigated, and systematic variation of material ratios remains an important area for future study. Raman spectroscopy and mechanical testing were presented primarily as qualitative or representative analyses; quantitative evaluation across multiple samples would further strengthen the interpretation of matrix development and construct evolution. Swelling behaviour was reported descriptively due to the absence of replicate-level raw data, precluding statistical comparison. Live/Dead imaging provides information on surface-level cell viability but does not fully capture spatial distribution throughout the 3D constructs.

Finally, although transcriptional analysis indicates early-stage osteogenic commitment, additional functional validation—such as protein expression, alkaline phosphatase activity, and matrix mineralisation—is required to confirm full differentiation. Further studies incorporating immunological assessment and in vivo models will also be necessary to support clinical translation.

## 3. Conclusions

To summarise, composite hydrogels composed of gelatin and alginate were evaluated as cell-laden bioinks for extrusion-based 3D bioprinting. The hydrogels exhibited suitable physicochemical properties and supported cell viability in vitro. Rapid hydration was observed following extrusion, followed by gradual degradation over three weeks, likely due to ion exchange-mediated instability and partial dissolution of gelatin.

Encapsulated hBM-MSCs remained viable, confirming the cytocompatibility of the hydrogel system for 3D culture. Under osteogenic conditions, changes in metabolic activity and upregulation of RUNX2, COL1A1, and OCN were observed, indicating early commitment towards an osteogenic lineage. However, as no protein-level or functional assays were performed, these findings should be interpreted as evidence of early differentiation at the transcriptional level.

Overall, this study provides a baseline physicochemical and biological characterisation of a commonly used gelatin–alginate bioink under standardised bioprinting conditions. These findings offer a reference for future studies aimed at optimising bioink formulations and evaluating functional performance in bone tissue engineering applications.

## 4. Materials and Methods

### 4.1. Hydrogel Preparation

The gelatin–alginate formulation used in this study (3% *w*/*v* gelatin and 5% *w*/*v* sodium alginate) was selected based on previous work demonstrating suitable printability and structural stability in extrusion-based 3D bioprinted hydrogel scaffolds for osteochondral applications [33]. This composition provides a balance between gelatin-mediated cell-interactive properties and alginate-driven ionic crosslinking, enabling stable construct formation following Ca^2+^ crosslinking. Sodium alginate (5% *w*/*v*; Sigma-Aldrich, Merck KGaA, Darmstadt, Germany) and gelatin (3% *w*/*v*; porcine skin type A, ~300 Bloom; Sigma-Aldrich, Merck KGaA, Darmstadt, Germany) were prepared in sterile cell culture medium at 55 °C under magnetic stirring (150 rpm) until fully dissolved. The solutions were centrifuged (200 rpm, 5 min) to remove entrapped air bubbles and stored at 4 °C until use. Prior to bioprinting, hydrogel stocks were warmed to 37 °C to restore suitable viscosity for mixing and extrusion.

### 4.2. Cell Culture and Differentiation Conditions

Human bone marrow–derived mesenchymal stem cells (hBM-MSCs) were purchased from PromoCell GmbH (Heidelberg, Germany) and cultured according to the supplier’s instructions. Cells were expanded in MSC Growth Medium (PromoCell GmbH) supplemented with the recommended growth supplements and maintained at 37 °C in a humidified atmosphere containing 5% CO_2_. Culture medium was changed every 2–3 days, and cells were passaged upon reaching approximately 80–90% confluence. Cells at passages 3–5 were used for all experiments.

For lineage-specific differentiation studies, cell-laden constructs were cultured in either standard MSC growth medium (control), osteogenic differentiation medium, or chondrogenic differentiation medium. The osteogenic medium consisted of Dulbecco’s Modified Eagle Medium (DMEM) supplemented with 10% foetal bovine serum (FBS), 1% penicillin–streptomycin, 10 mM β-glycerophosphate, 50 µg/mL L-ascorbic acid, and 100 nM dexamethasone.

For chondrogenic induction, constructs were cultured in chondrogenic differentiation medium (PromoCell GmbH) according to the manufacturer’s protocol. Media were refreshed every 2–3 days throughout the culture period. Chondrogenic differentiation was not evaluated at the gene expression level in the present study and was included to model lineage-specific bioink conditions for physicochemical comparison.

### 4.3. Extrusion-Based 3D Bioprinting of Bone Scaffolds

Bioprinting was performed using a Regemat3D extrusion bioprinter (Granada, Spain) with 0.41 mm tips. Constructs (12 mm diameter, 2.1 mm height, 0.2 mm pore size, 0.35 mm fibre width) were printed at 32 °C and 6 mm/s. For cell-laden formulations, human bone marrow-derived mesenchymal stem cells (hBM-MSCs) were suspended in the hydrogel precursor at a density of 5 × 10^6^ cells/mL under sterile conditions. The cell–polymer mixture was gently homogenised to achieve uniform cell distribution while minimising shear-induced cellular damage.

Following extrusion-based 3D bioprinting, constructs were ionically crosslinked by immersion in 100 mM calcium chloride (CaCl_2_) solution for 15–20 min at room temperature to stabilise the alginate network (Figure S1). Crosslinked constructs were rinsed with sterile PBS to remove excess calcium ions before subsequent experiments. All printing experiments were performed in triplicate to ensure reproducibility.

For osteogenic induction relevant to bone regeneration, constructs were cultured either in standard MSC growth medium or osteogenic differentiation medium consisting of DMEM supplemented with 10% FBS, 1% penicillin–streptomycin, 10 mM β-glycerophosphate, 50 µg/mL L-ascorbic acid, and 100 nM dexamethasone.

### 4.4. Water Uptake Percentage

Water uptake percentage was evaluated by immersing lyophilised scaffolds (initial weight, W_0_) in PBS at 37 °C. At predetermined time points (1, 4, 8, and 24 h), samples were removed, gently blotted to eliminate surface moisture, and weighed (W_t_). The ratio was calculated using the following equation:

$$\mathrm{Water}\;\mathrm{Uptake}\;\mathrm{Percentage}\;=\;\frac{Wt-Wo\;}{W0}\times 100$$

### 4.5. Degradation Study

To evaluate degradation under physiological conditions relevant to bone remodelling, scaffolds were incubated in PBS (hydrolytic degradation) or 0.16 mg/mL collagenase Type II solution at 37 °C. Samples were collected at 1, 4, 7, 10, 14, and 21 days. After removal from the degradation medium, constructs were frozen at −20 °C, lyophilised, and weighed (W_t_). The percentage of mass remaining was calculated as:

$$\mathrm{Degradation}\;\mathrm{Rate}\;(\%)\;=\;\frac{W0-Wt\;}{Wo}\times 100$$

All measurements were conducted in triplicate.

### 4.6. Cell Viability and Metabolic Activity

Cell viability within the printed constructs was assessed using the alamarBlue assay according to the manufacturer’s protocol. At designated time points (Days 1, 4, and 7), culture medium was replaced with fresh medium containing 10% (*v*/*v*) alamarBlue reagent and incubated at 37 °C for the recommended duration. Following incubation, aliquots were transferred to a 96-well plate, and absorbance was measured at 570 nm and 600 nm using a microplate reader. Background readings from cell-free hydrogel controls were subtracted. Metabolic activity was expressed as optical density (OD). Experiments were performed in triplicate.

Live/Dead staining was conducted after 24 h of culture. Constructs were incubated in staining solution containing 4 mM Calcein-AM and 2 mM ethidium homodimer-1 for 60 min at 37 °C and imaged using an Olympus IX51 epifluorescence microscope (Olympus, Tokyo, Japan).

### 4.7. Scanning Electron Microscopy (SEM)

The microstructure and cellular morphology within scaffolds were examined by qualitative scanning electron microscopy (SEM). For structural analysis, printed scaffolds were frozen at −80 °C and lyophilised for 24 h. Samples were mounted on aluminium stubs using conductive carbon tape and sputter-coated with a thin layer (~10 nm) of gold using a sputter coater (Quorum Technologies, Lewes, UK).

For cell morphology evaluation, hBM-MSC-laden constructs were cultured under control (growth medium) or osteogenic conditions and analysed at Day 7. Constructs were fixed in 2.5% glutaraldehyde in PBS for 2 h at room temperature, followed by graded ethanol dehydration (30%, 50%, 70%, 90%, and 100%). Samples were dried, mounted, sputter-coated, and imaged using a scanning electron microscope (JEOL JSM-IT100, JEOL Ltd., Tokyo, Japan) at an accelerating voltage of 5–10 kV.

### 4.8. RNA Extraction and Quantitative Real-Time PCR

To evaluate osteogenic differentiation within the bioprinted constructs, gene expression analysis was performed at Day 14 (and other specified time points if applicable). Total RNA was extracted using TRIzol reagent (Thermo Fisher Scientific, Waltham, MA, USA) according to the manufacturer’s instructions. RNA concentration and purity were determined using a NanoDrop spectrophotometer (Thermo Fisher Scientific), and samples with an A260/A280 ratio between 1.8 and 2.0 were used for further analysis.

Complementary DNA (cDNA) was synthesised from 1 µg of total RNA using a reverse transcription kit (Applied Biosystems, Foster City, CA, USA) following the manufacturer’s protocol.

Quantitative real-time PCR (qPCR) was performed using SYBR iTaq Universal SYBR Green Supermix (Bio-Rad Laboratories, Hercules, CA, USA) on a real-time PCR detection system (Bio-Rad). The osteogenic marker genes RUNX2, COL1A1, and osteocalcin (OCN) were analysed (Table S1). RPL-19 was used as the internal reference gene. Each reaction was performed in triplicate.

Relative gene expression levels were calculated using the 2^−ΔΔCt^ method. To improve normality for statistical analysis and graphical presentation, fold-change values were log10-transformed before statistical testing. Results are presented as log10 (fold change) relative to the baseline control condition.

### 4.9. Raman Spectrometry

Raman spectra of bioprinted hydrogel constructs (with or without encapsulated cells) were acquired using a confocal Raman microscope equipped with a 532 nm excitation laser. Spectra were collected over the range of 600–1800 cm^−1^ to capture characteristic biomolecular vibrations. Raw spectra were processed using MATLAB R2019 (MathWorks, Natick, MA, USA), including baseline correction, smoothing, and normalisation to minimise background noise and enable comparison between samples. Peak assignments were performed based on previously reported literature. Spectra were obtained at defined culture time points (Day 3, Day 14, and Day 21 for cell-laden constructs; Day 14 for acellular controls) and are presented as representative measurements.

### 4.10. Mechanical Testing

The compressive mechanical properties of hydrogel constructs were evaluated using an Instron 5967 Universal Testing Machine (Instron, Norwood, MA, USA) under unconfined compression. Samples were compressed at a constant strain rate of approximately 25% strain per minute until the desired deformation was reached. Stress–strain curves were recorded, and the compressive Young’s modulus (E = σ/ε) was determined from the initial linear elastic region of the curve. This analysis was used to compare the bulk stiffness of printed and non-printed hydrogel constructs, as well as the influence of cell encapsulation on mechanical behaviour. Acellular (non-cell) hydrogel constructs were prepared using the same gelatin–alginate formulation without the addition of cells. These samples were bioprinted and crosslinked under identical conditions as the cell-laden constructs and served as controls to assess the effect of cell encapsulation on mechanical behaviour.

### 4.11. Statistical Analysis

Statistical analyses were performed using SPSS software (Version 31.0.0.0, IBM Corp., Armonk, NY, USA). Data are presented as mean ± standard deviation (SD). For all datasets, normality and homogeneity of variance were assessed using Shapiro–Wilk and Levene’s tests, respectively. For degradation studies, three-way ANOVA was conducted to evaluate the effects of bioink type, degradation medium (PBS vs collagenase), and time, followed by Bonferroni post hoc testing where appropriate. For cell viability (proliferation) data, which involved longitudinal measurements, a two-way mixed ANOVA was employed. Due to the violation of the assumption of sphericity (Mauchly’s test, *p* < 0.001), degrees of freedom were adjusted using the Greenhouse–Geisser correction. Furthermore, as Levene’s test indicated unequal variances across groups (*p* < 0.05), the Games–Howell post hoc test was utilised for pairwise comparisons, as it is robust against heteroscedasticity. For gene expression analysis, data were log-transformed (LN) to satisfy the assumptions of parametric testing. Following transformation, the assumption of homogeneity of variance was met (Levene’s test, *p* > 0.05). Consequently, a standard ANOVA followed by Bonferroni post hoc correction was applied for multiple comparisons to maintain strict control over Type I error rates. Significance for all tests was set at *p* < 0.05.

## Data Availability

The data presented in this study are available from the corresponding author upon reasonable request.

## Supplementary Materials

The following supporting information can be downloaded at: https://www.mdpi.com/article/10.3390/gels12050387/s1, Table S1. Details of housekeeping genes targeted by qPCR amplification. Figure S1. Representative images of 3D bioprinted gelatin–alginate constructs before and after ionic crosslinking. (A) Immediately after printing, prior to crosslinking, constructs exhibit less defined morphology and reduced structural integrity. (B) Following ionic crosslinking, constructs display improved shape fidelity and structural stability, forming well-defined hydrogel constructs.

## Abbreviations

The following abbreviations are used in this manuscript:

hBM-MSC	Human Bone Marrow-Derived Mesenchymal Stem Cells
3D	Three dimensional
SEM	Scanning Electron Microscopy
HOB	Human Osteoblasts
COL1A1	Collagen Type 1Alpha 1 Chain
OCN	Osteocalcin
Ct	Cycle threshold
DMEM	Dulbecco’s Modified Eagle Medium
FBS	Foetal Bovine Serum
PBS	Phosphate-Buffered Saline
qPCR	Quantitative Polymerase Chain Reaction
RNA	Ribonucleic Acid
CaCl_2_	Calcium Chloride

## References

[1] Wu A.-M., Bisignano C., James S.L., Abady G.G., Abedi A., Abu-Gharbieh E., Alhassan R.K., Alipour V., Arabloo J., Asaad M. (2021). Global, regional, and national burden of bone fractures in 204 countries and territories, 1990–2019: A systematic analysis from the Global Burden of Disease Study 2019. Lancet Healthy Longev..

[2] Oryan A., Alidadi S., Moshiri A., Maffulli N. (2014). Bone regenerative medicine: Classic options, novel strategies, and future directions. J. Orthop. Surg. Res..

[3] Karuppal R. (2017). Current concepts in the articular cartilage repair and regeneration. J. Orthop..

[4] Amini A.R., Laurencin C.T., Nukavarapu S.P. (2012). Bone tissue engineering: Recent advances and challenges. Crit. Rev. Biomed. Eng..

[5] Henkel J., Woodruff M.A., Epari D.R., Steck R., Glatt V., Dickinson I.C., Choong P.F., Schuetz M.A., Hutmacher D.W. (2013). Bone regeneration based on tissue engineering conceptions—A 21st century perspective. Bone Res..

[6] Alonzo M., Primo F.A., Kumar S.A., Mudloff J.A., Dominguez E., Fregoso G., Ortiz N., Weiss W.M., Joddar B. (2021). Bone tissue engineering techniques, advances, and scaffolds for treatment of bone defects. Curr. Opin. Biomed. Eng..

[7] Zhang J., Wehrle E., Rubert M., Müller R. (2021). 3D bioprinting of human tissues: Biofabrication, bioinks, and bioreactors. Int. J. Mol. Sci..

[8] Ong C.S., Yesantharao P., Huang C.Y., Mattson G., Boktor J., Fukunishi T., Zhang H., Hibino N. (2018). 3D bioprinting using stem cells. Pediatr. Res..

[9] Zhang Y.S., Haghiashtiani G., Hübscher T., Kelly D.J., Lee J.M., Lutolf M., McAlpine M.C., Yeong W.Y., Zenobi-Wong M., Malda J. (2021). 3D extrusion bioprinting. Nat. Rev. Methods Primers.

[10] Khajehmohammadi M., Bakhtiary N., Davari N., Sarkari S., Tolabi H., Li D., Ghalandari B., Yu B., Ghorbani F. (2023). Bioprinting of cell-laden protein-based hydrogels: From cartilage to bone tissue engineering. Int. J. Bioprint..

[11] Labowska M.B., Cierluk K., Jankowska A.M., Kulbacka J., Detyna J., Michalak I. (2021). A Review on the Adaption of Alginate-Gelatin Hydrogels for 3D Cultures and Bioprinting. Materials.

[12] Yan Y., Wang X., Pan Y., Liu H., Cheng J., Xiong Z., Lin F., Wu R., Zhang R., Lu Q. (2005). Fabrication of viable tissue-engineered constructs with 3D cell-assembly technique. Biomaterials.

[13] Yan Y., Wang X., Xiong Z., Liu H., Liu F., Lin F., Wu R., Zhang R., Lu Q. (2005). Direct construction of a three-dimensional structure with cells and hydrogel. J. Bioact. Compat. Polym..

[14] Wang X., Yan Y., Pan Y., Xiong Z., Liu H., Cheng J., Liu F., Lin F., Wu R., Zhang R. (2006). Generation of three-dimensional hepatocyte/gelatin structures with rapid prototyping system. Tissue Eng..

[15] Li S., Xiong Z., Wang X., Yan Y., Liu H., Zhang R. (2009). Direct fabrication of a hybrid cell/hydrogel construct by a double-nozzle assembling technology. J. Bioact. Compat. Polym..

[16] Xu M., Wang X., Yan Y., Yao R., Ge Y. (2010). An cell-assembly derived physiological 3D model of the metabolic syndrome, based on adipose-derived stromal cells and a gelatin/alginate/fibrinogen matrix. Biomaterials.

[17] Xu M., Van Y., Liu H., Yag R., Wang X. (2009). Controlled adipose-derived stromal cells differentiation into adipose and endothelial cells in a 3D structure established by cell-assembly technique. J. Bioact. Compat. Polym..

[18] Wang X., Ao Q., Tian X., Fan J., Tong H., Hou W., Bai S. (2017). Gelatin-based hydrogels for organ 3D bioprinting. Polymers.

[19] Lee K.Y., Mooney D.J. (2001). Hydrogels for tissue engineering. Chem. Rev..

[20] Naghizadeh Z., Karkhaneh A., Khojasteh A. (2018). Self-crosslinking effect of chitosan and gelatin on alginate based hydrogels: Injectable in situ forming scaffolds. Mater. Sci. Eng. C.

[21] Lee K.Y., Mooney D.J. (2012). Alginate: Properties and biomedical applications. Prog. Polym. Sci..

[22] Bidarra S.J., Barrias C.C., Granja P.L. (2014). Injectable alginate hydrogels for cell delivery in tissue engineering. Acta Biomater..

[23] Sonaye S.Y., Ertugral E.G., Kothapalli C.R., Sikder P. (2022). Extrusion 3D (bio) printing of alginate-gelatin-based composite scaffolds for skeletal muscle tissue engineering. Materials.

[24] Maisani M., Pezzoli D., Chassande O., Mantovani D. (2017). Cellularizing hydrogel-based scaffolds to repair bone tissue: How to create a physiologically relevant micro-environment?. J. Tissue Eng..

[25] Langer R., Peppas N.A. (2003). Advances in biomaterials, drug delivery, and bionanotechnology. AIChE J..

[26] Tajvar S., Hadjizadeh A., Samandari S.S. (2023). Scaffold degradation in bone tissue engineering: An overview. Int. Biodeterior. Biodegrad..

[27] Lutolf M.P., Hubbell J. (2005). Synthetic biomaterials as instructive extracellular microenvironments for morphogenesis in tissue engineering. Nat. Biotechnol..

[28] Li Z.-Y., Li T.-Y., Yang H.-C., Ding M.-H., Chen L.-J., Yu S.-Y., Meng X.-S., Jin J.-J., Sun S.-Z., Zhang J. (2024). Design and fabrication of viscoelastic hydrogels as extracellular matrix mimicry for cell engineering. Chem Bio Eng..

[29] Pittenger M.F., Mackay A.M., Beck S.C., Jaiswal R.K., Douglas R., Mosca J.D., Moorman M.A., Simonetti D.W., Craig S., Marshak D.R. (1999). Multilineage potential of adult human mesenchymal stem cells. Science.

[30] Komori T. (2018). Runx2, an inducer of osteoblast and chondrocyte differentiation. Histochem. Cell Biol..

[31] Bruderer M., Richards R., Alini M., Stoddart M.J. (2014). Role and regulation of RUNX2 in osteogenesis. Eur. Cells Mater..

[32] Choe G., Lee M., Oh S., Seok J.M., Kim J., Im S., Park S.A., Lee J.Y. (2022). Three-dimensional bioprinting of mesenchymal stem cells using an osteoinductive bioink containing alginate and BMP-2-loaded PLGA nanoparticles for bone tissue engineering. Biomater. Adv..

[33] Chen H., Gonnella G., Huang J., Di-Silvio L. (2023). Fabrication of 3D bioprinted bi-phasic scaffold for bone–cartilage interface regeneration. Biomimetics.

[34] Dutta S.D., Hexiu J., Patel D.K., Ganguly K., Lim K.-T. (2021). 3D-printed bioactive and biodegradable hydrogel scaffolds of alginate/gelatin/cellulose nanocrystals for tissue engineering. Int. J. Biol. Macromol..

[35] Boucard E., Vidal L., Coulon F., Mota C., Hascoët J.-Y., Halary F. (2022). The degradation of gelatin/alginate/fibrin hydrogels is cell type dependent and can be modulated by targeting fibrinolysis. Front. Bioeng. Biotechnol..

[36] Liu C., Qin W., Wang Y., Ma J., Liu J., Wu S., Zhao H. (2021). 3D printed gelatin/sodium alginate hydrogel scaffolds doped with nano-attapulgite for bone tissue repair. Int. J. Nanomed..

[37] Wierzbicka A., Bartniak M., Waśko J., Kolesińska B., Grabarczyk J., Bociaga D. (2024). The impact of gelatin and fish collagen on alginate hydrogel properties: A comparative study. Gels.

[38] Karoyo A.H., Wilson L.D. (2021). A review on the design and hydration properties of natural polymer-based hydrogels. Materials.

[39] Torres M.L., Oberti T.G., Fernández J.M. (2020). HEMA and alginate-based chondrogenic semi-interpenetrated hydrogels: Synthesis and biological characterization. J. Biomater. Sci. Polym. Ed..

[40] Langenbach F., Handschel J. (2013). Effects of dexamethasone, ascorbic acid and β-glycerophosphate on the osteogenic differentiation of stem cells in vitro. Stem Cell Res. Ther..

[41] Mazzoni E., Mazziotta C., Iaquinta M.R., Lanzillotti C., Fortini F., D’Agostino A., Trevisiol L., Nocini R., Barbanti-Brodano G., Mescola A. (2021). Enhanced osteogenic differentiation of human bone marrow-derived mesenchymal stem cells by a hybrid hydroxylapatite/collagen scaffold. Front. Cell Dev. Biol..

[42] Sigmarsdottir T.B., McGarrity S., de Lomana A.L.G., Kotronoulas A., Sigurdsson S., Yurkovich J.T., Rolfsson O., Sigurjonsson O.E. (2021). Metabolic and transcriptional changes across osteogenic differentiation of mesenchymal stromal cells. Bioengineering.

[43] Müller W.E., Schröder H.C., Wang X. (2019). Inorganic polyphosphates as storage for and generator of metabolic energy in the extracellular matrix. Chem. Rev..

[44] Hu X., Xia Z., Cai K. (2022). Recent advances in 3D hydrogel culture systems for mesenchymal stem cell-based therapy and cell behavior regulation. J. Mater. Chem. B.

[45] Huang X., Huang Z., Gao W., Gao W., He R., Li Y., Crawford R., Zhou Y., Xiao L., Xiao Y. (2022). Current advances in 3D dynamic cell culture systems. Gels.

[46] Turnbull G., Clarke J., Picard F., Riches P., Jia L., Han F., Li B., Shu W. (2018). 3D bioactive composite scaffolds for bone tissue engineering. Bioact. Mater..

[47] Kim J., Choi Y.-J., Gal C.-W., Sung A., Park H., Yun H.-S. (2023). Development of an alginate-gelatin bioink enhancing osteogenic differentiation by gelatin release. Int. J. Bioprint..

[48] Stein G.S., Lian J.B., Stein J.L., Van Wijnen A.J., Montecino M. (1996). Transcriptional control of osteoblast growth and differentiation. Physiol. Rev..

[49] Movasaghi Z., Rehman S., Rehman I.U. (2007). Raman spectroscopy of biological tissues. Appl. Spectrosc. Rev..

[50] Talari A.C.S., Movasaghi Z., Rehman S., Rehman I.U. (2015). Raman spectroscopy of biological tissues. Appl. Spectrosc. Rev..

[51] Bergholt M.S., Serio A., Albro M.B. (2019). Raman Spectroscopy: Guiding Light for the Extracellular Matrix. Front. Bioeng. Biotechnol..

[52] Mandair G.S., Morris M.D. (2015). Contributions of Raman spectroscopy to the understanding of bone strength. BoneKEy Rep..

[53] Unal M. (2021). Raman spectroscopic determination of bone matrix quantity and quality augments prediction of human cortical bone mechanical properties. J. Biomech..

[54] Litasov K.D., Podgornykh N.M. (2017). Raman spectroscopy of various phosphate minerals and occurrence of tuite in the Elga IIE iron meteorite. J. Raman Spectrosc..

[55] Hashemnejad S.M., Kundu S. (2016). Strain stiffening and negative normal stress in alginate hydrogels. J. Polym. Sci. Part B Polym. Phys..

[56] Goudoulas T.B., Didonaki A., Pan S., Fattahi E., Becker T. (2023). Comparative large amplitude oscillatory shear (LAOS) study of ionically and physically crosslinked hydrogels. Polymers.

[57] Dastgerdi J.N., Koivisto J.T., Orell O., Rava P., Jokinen J., Kanerva M., Kellomäki M. (2021). Comprehensive characterisation of the compressive behaviour of hydrogels using a new modelling procedure and redefining compression testing. Mater. Today Commun..

[58] Jeon O., Kim T.-H., Alsberg E. (2021). Reversible dynamic mechanics of hydrogels for regulation of cellular behavior. Acta Biomater..

[59] Rosales A.M., Anseth K.S. (2016). The design of reversible hydrogels to capture extracellular matrix dynamics. Nat. Rev. Mater..

[60] Zhang Y., Wang Z., Sun Q., Li Q., Li S., Li X. (2023). Dynamic hydrogels with viscoelasticity and tunable stiffness for the regulation of cell behavior and fate. Materials.

[61] Engler A.J., Sen S., Sweeney H.L., Discher D.E. (2006). Matrix elasticity directs stem cell lineage specification. Cell.

[62] Chaudhuri O., Cooper-White J., Janmey P.A., Mooney D.J., Shenoy V.B. (2020). Effects of extracellular matrix viscoelasticity on cellular behaviour. Nature.

[63] Virdi J.K., Pethe P. (2021). Biomaterials regulate mechanosensors YAP/TAZ in stem cell growth and differentiation. Tissue Eng. Regen. Med..

