# Supplementary material for: hBM-MSC-Laden 3D Bioprinted Gelatin–Alginate Hydrogels: Physicochemical Characterisation and Osteogenic Lineage Commitment
Source: Gels. 2026 May 1;12(5):387. doi: 10.3390/gels12050387 (PMC13205829; doi:10.3390/gels12050387)
Supplement: Supplementary file 1 [file gels-12-00387-s001.zip › gels-4197862-supplementary.pdf]

Supplementary Materials:

Table S1. Details of housekeeping and target genes used for qPCR amplification.

	Manufacturer	Primer sequence (5′-3')
RUNX2	Sigma-Aldrich<br>SY130325134-021	F-CCA ACC CAC GAA TGC ACT ATC
	Sigma-Aldrich<br>SY130325134-022	R-TAG TGA GTG GTG GCG GAC ATA
RPL-19	Sigma-Aldrich<br>SY140324324-053	F-CAG AAG ATA CCG TGA ATC T
	Sigma-Aldrich<br>SY140324324-054	R-TGT TTT TGA ACA CAT T
Collagen IA1	IDT<br>477921711	F-GAT TCC CTG GAC CTA AAG GTG C
	IDT<br>477921712	R-AGC CTC TCC ATC TTT GCC AGC A
Osteocalcin	Sigma-Aldrich<br>SY110614664-015	F-CTC ACA CTC CTC GCC CTA TT
	Sigma-Aldrich<br>SY110707219-006	R-AGG GGA AGA GGA AAG AAG

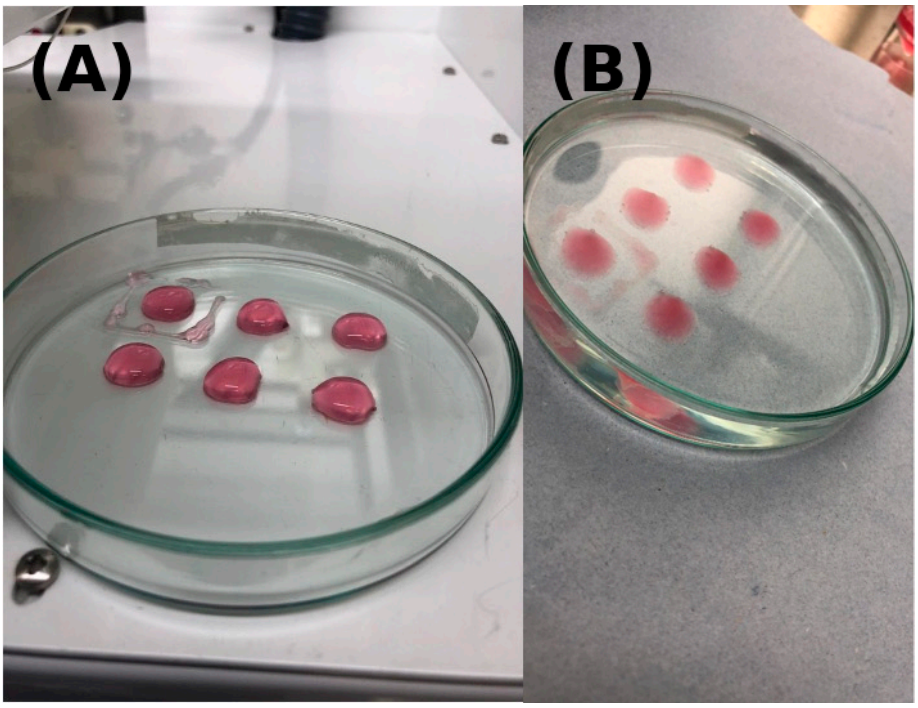

Supplementary Figure S1. Representative images of 3D bioprinted gelatin–alginate constructs before and after ionic crosslinking. (A) Immediately after printing, prior to crosslinking, constructs exhibit less defined morphology and reduced structural integrity. (B) Following ionic crosslinking, constructs display improved shape fidelity and structural stability, forming well-defined hydrogel constructs.
